# Prophylaxis with intrathecal or high-dose methotrexate in diffuse large B-cell lymphoma and high risk of CNS relapse

**DOI:** 10.1038/s41408-021-00506-3

**Published:** 2021-06-16

**Authors:** Sabela Bobillo, Erel Joffe, David Sermer, Patrizia Mondello, Paola Ghione, Philip C. Caron, Audrey Hamilton, Paul A. Hamlin, Steven M. Horwitz, Anita Kumar, Matthew J. Matasar, Connie L. Batlevi, Alison Moskowitz, Ariela Noy, Collette N. Owens, M. Lia Palomba, David Straus, Gottfried von Keudell, Ahmet Dogan, Andrew D. Zelenetz, Venkatraman E. Seshan, Anas Younes

**Affiliations:** 1 Department of Medicine, Lymphoma Service, Memorial Sloan Kettering Cancer Center, New York, NY USA; 2 Department of Hematology, Vall d’Hebron Institute of Oncology (VHIO), Barcelona, Spain; 3 Department of Medicine, Universitat Autonoma de Barcelona, Barcelona, Spain; 4 Weill Cornell Department of Medicine, Weill Cornell Medical College, New York, NY USA; 5 Department of Pathology, Memorial Sloan Kettering Cancer Center, New York, NY USA; 6 Department of Epidemiology and Biostatistics, Memorial Sloan Kettering Cancer Center, New York, NY USA

**Keywords:** B-cell lymphoma, Disease-free survival

## Abstract

Although methotrexate (MTX) is the most widely used therapy for central nervous system (CNS) prophylaxis in patients with diffuse large B-cell lymphoma (DLBCL), the optimal regimen remains unclear. We examined the efficacy of different prophylactic regimens in 585 patients with newly diagnosed DLBCL and high-risk for CNS relapse, treated with rituximab, cyclophosphamide, doxorubicin, vincristine, and prednisone (R-CHOP) or R-CHOP-like regimens from 2001 to 2017, of whom 295 (50%) received prophylaxis. Intrathecal (IT) MTX was given to 253 (86%) and high-dose MTX (HD-MTX) to 42 (14%). After a median follow-up of 6.8 years, 36 of 585 patients relapsed in the CNS, of whom 14 had received prophylaxis. The CNS relapse risk at 1 year was lower for patients who received prophylaxis than patients who did not: 2% vs. 7.1%. However, the difference became less significant over time (5-year risk 5.6% vs. 7.5%), indicating prophylaxis tended to delay CNS relapse rather than prevent it. Furthermore, the CNS relapse risk was similar in patients who received IT and HD-MTX (5-year risk 5.6% vs. 5.2%). Collectively, our data indicate the benefit of MTX for CNS prophylaxis is transient, highlighting the need for more effective prophylactic regimens. In addition, our results failed to demonstrate a clinical advantage for the HD-MTX regimen.

## Introduction

Diffuse large B-cell lymphoma (DLBCL) is the most common subtype of lymphoma accounting for 30–40% of all non-Hodgkin lymphomas. Central nervous system (CNS) relapse is an uncommon yet often fatal complication with a median overall survival (OS) of less than 6 months [1]. Overall, the incidence of CNS relapse in patients with DLBCL is around 2%, which is lower than with other aggressive lymphomas, such as Burkitt lymphoma or acute lymphoblastic leukemia. However, the presence of certain risk factors might increase the risk of CNS relapse to 15% [2].

Models have been made to identify patients with high risk of CNS relapse. The German High-Grade non-Hodgkin Lymphoma Study Group (DSHNHL) recently proposed a CNS prognostic model (CNS-IPI) that includes five international prognostic index (IPI) factors and the involvement of kidney or adrenal glands. This model stratified DLBCL patients into three categories, low (0–1 risk factors), intermediate (2–3 risk factors), and high risk (4–6 risk factors) with a 2-year rate of CNS relapse of 0.6%, 3.4%, and 10.2%, respectively [3]. However, involvement of certain extranodal sites, such as testes, breast, or bone marrow also confers an increased risk, even with low CNS-IPI [4–7]. In addition, the presence of *MYC* translocation together with *BCL2* translocation has been also associated with a higher risk of CNS relapse in several retrospective series [8, 9]. Finally, the combination of cell of origin (COO) determined by gene expression profiling (GEP) and CNS-IPI has recently improved the identification of DLBCL patients with high risk of CNS relapse, showing a 2-year CNS relapse rate up to 15% in patients with activated B-cell (ABC) phenotype and high CNS-IPI [10].

In high-risk patients, CNS prophylaxis is usually recommended, although the optimal regimen remains unclear. Some prospective and retrospective studies conducted in the rituximab era have demonstrated the lack of efficacy of intrathecal (IT) methotrexate (MTX) in patients treated with rituximab, cyclophosphamide, doxorubicin, vincristine, and prednisone (R-CHOP) or similar regimens [2, 10–13]. High-dose intravenous methotrexate (HD-MTX) has been postulated as a possibly better option since the majority of relapses in the rituximab era occur in the brain parenchyma. However, different retrospective studies have shown conflicting results regarding its efficacy [14–19]. Finally, preliminary results from a multicenter retrospective study showed similar effectiveness of prophylactic HD-MTX and IT MTX in patients with aggressive non-Hodgkin lymphoma [20].

The purpose of this study was to evaluate the efficacy of different CNS prophylaxis regimens in preventing CNS relapse in DLBCL patients with risk factors for CNS recurrence who were treated with rituximab and chemotherapy in a single institution.

## Patients and methods

### Patients

We retrospectively reviewed the records of all newly diagnosed patients with DLBCL at Memorial Sloan Kettering Cancer Center (MSKCC) from 2001 to 2017, treated with frontline R-CHOP or R-CHOP-like regimens. In all cases, the pathology at diagnosis was confirmed by expert hematopathologists at MSKCC according to the World Health Organization classification of hematopoietic and lymphoid tumors. Patients with primary mediastinal B cell lymphoma, HIV positive or known CNS disease at diagnosis were excluded. Patients with history of indolent lymphoma previously treated with chemotherapy were also excluded. The Hans algorithm [21] was used to classify patients as germinal center B-cell like phenotype (GCB) or non-germinal center B-cell like (non-GCB). High risk for CNS relapse (HR-CNS) was defined by high-CNS-IPI (4–6 risk factors) or low or intermediate CNS-IPI along with testicular, breast, kidney, adrenal glands, and/or bone marrow involvement. Patients with *MYC* and *BCL2* rearrangement were also included in HR-CNS group. Patients with low or intermediate CNS-IPI and paraspinal masses, sinus, orbit or skull involvement were not considered HR-CNS.

CNS prophylaxis was administered based on physician’s preference. CNS relapse was diagnosed by the presence of radiological findings, detection of lymphoma cells in the CSF and/or by brain biopsy. This study was approved by the institutional reviewed board at MSKCC.

### Statistical analysis

Competing risk analysis was used to analyze time to CNS relapse, with systemic non-CNS relapse and death without relapse as competing events. Time to event was calculated from the date of diagnosis to the date of one of the three events, relapse in the CNS, systemic relapse, or death without relapse whichever occurs first. OS from relapse was defined as the time from relapse until death of any cause. Patients’ characteristics were compared using Fisher exact test for categorical variables and Kruskal–Wallis test for continuous variables. Kaplan-Meier analysis was implemented to report survival estimates across patients and logrank test to compare groups. We applied a non-parametric analysis of competing risks and used Gray’s test to compare the cumulative incidence of CNS relapse between the different groups, with death and systemic relapse as competing events. Confidence intervals for risk ratio (RR) (ratio of cumulative incidence rate at fixed time-points) were obtained using bootstrap resampling. All statistical analyses were performed in R v3.6.

## Results

### Patient characteristics

**Fig. 1:**
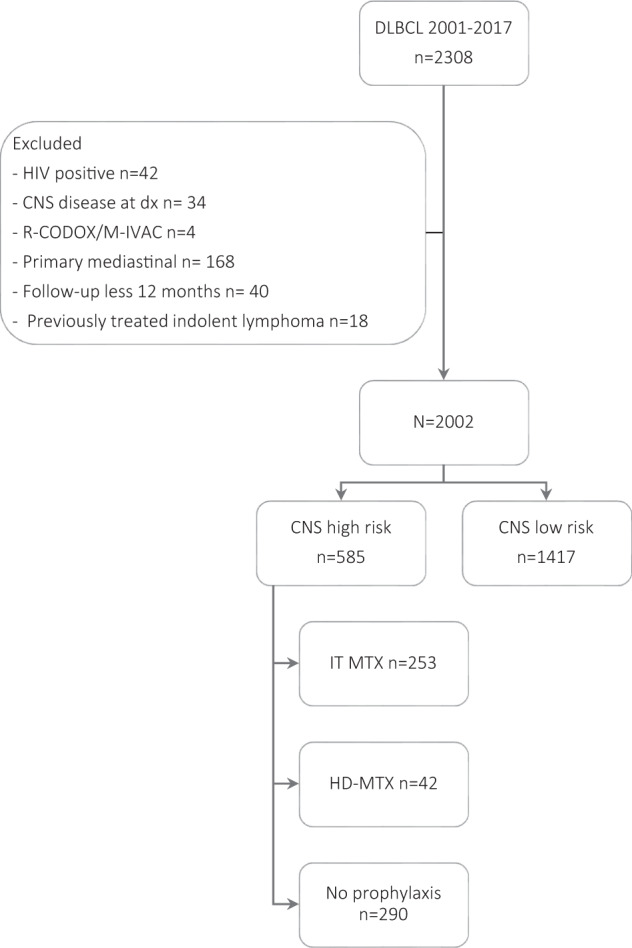
Consort diagram. DLBCL diffuse large B cell lymphoma, CNS central nervous system, IT MTX intrathecal methotrexate, HD-MTX high-dose methotrexate.

We identified 2308 patients treated with R-CHOP or R-CHOP-like regimens from 2001 to 2017 at MSKCC. We excluded 306 patients for the following reasons: HIV positive, *n* = 42, CNS disease at diagnosis, *n* = 34; primary mediastinal lymphoma, *n* = 168; previously treated indolent lymphoma, *n* = 18; treatment with R-CODOX/M-IVAC, *n* = 4 and follow-up shorter than 12 months, *n* = 40. Finally, 2002 patients were considered and 585 (29%) were classified as HR-CNS relapse and were included in the study (Fig. 1).

Median age was 68 years (range 21–91) and 301 (51%) patients were male. The high-risk extranodal sites were: BM, *n* = 118 (20%), kidney/adrenal, *n* = 106 (18%); testes, *n* = 51 (9%) and breast, *n* = 47 (8%). One hundred and fifty-eight patients (27%) had more than two extranodal sites involved. By CNS-IPI, patients were classified as low, 10%; intermediate, 22%; and high risk, 68%. Eighteen (7%) patients had *MYC* and *BCL2* rearrangements. COO determined by Hans algorithm [21] was non-germinal center (non-GCB) in 229 (39%) patients, germinal center (GCB) in 234 (40%) patients and missing in 122 (21%).

### CNS prophylaxis

Two hundred and ninety (50%) patients received at least one administration of CNS prophylaxis: IT MTX and/or cytarabine, *n* = 253 (87%); or HD-MTX, *n* = 42 (13%). Among the 42 patients who received HD-MTX, 11 received concomitant IT prophylaxis. Patients’ characteristics by prophylactic regimen are shown in Table 1. Patients who received prophylaxis (IT or HD-MTX) were younger (*p* < 0.001) and presented with a better performance status (ECOG < 2) (*p* = 0.002) compared with patients who did not receive prophylaxis. Patients with two or more extranodal sites involvement were more likely to receive CNS prophylaxis (*p* < 0.001).

**Table 1:** Patient’s characteristics.

Variables	IT methotrexate *n* (%)	HD-methotrexate *n* (%)	No prophylaxis *n* (%)	*p* value
Number (*n*)	253 (43%)	42 (7%)	290 (50%)	
Median age (range)	64 (21–86)	63 (27–81)	72 (24–91)	<0.001
Male	142 (56)	24 (57)	135 (47)	0.07
ECOG				
0–1	160 (63)	27 (64)	142 (49)	0.002
≥2	93 (37)	15 (38)	148 (51)	
Stage				
I–II	30 (12)	4 (10)	30 (10)	0.81
III–IV	223 (88)	38 (90)	260 (90)	
Serum LDH				
Above normal	198 (78)	28 (67)	207 (71)	0.14
Missing	5 (2)	0	17 (6)	
CNS-IPI risk^a^				
0	10 (4)	3 (7)	12 (4)	
1	16 (6)	2 (5)	18 (6)	
2	28 (11)	4 (10)	17 (6)	
3	41 (16)	4 (9)	31 (11)	
4	107 (43)	16 (38)	157 (54)	
5	41 (16)	12 (29)	49 (17)	
6	10 (4)	1 (2)	6 (2)	
CNS-IPI risk groups^a^				
Low 0–1	26 (10)	5 (12)	30 (10)	0.009
Intermediate 2–3	69 (27)	8 (19)	48 (17)	
High 4–6	158 (63)	29 (69)	212 (73)	
High risk site				
Testis	39 (15)	8 (19)	4 (1)	
Breast	15 (6)	5 (12)	27 (9)	
Kidney/adrenal glands	50 (20)	12 (29)	44 (15)	
Bone marrow	63 (25)	6 (14)	49 (17)	
Extranodal sites				
>2 sites	87 (36)	14 (35)	57 (20)	<0.001
Double-hit	18 (7)	5 (12)	11 (4)	0.05
Treatment				
R-CHOP	143 (57)	32 (76)	220 (76)	<0.001
R-EPOCH	43 (17)	7 (17)	40 (14)	
R-CHOP/RICE	67 (26)	3 (7)	30 (10)	
Cell of origin				
Germinal center	104 (41)	14 (33)	116 (40)	0.15
Non-germinal center	104 (41)	23 (55)	102 (35)	
Missing	45 (18)	5 (12)	72 (25)	

*IT* intrathecal, *MTX* methotrexate, *HD* high-dose, *ECOG* Eastern Cooperative Oncology Group, *LDH* lactate dehydrogenase, *CNS-IPI* central nervous system international prognostic index, *R-CHOP* rituximab, cyclophosphamide, doxorubicin, vincristine, prednisone, *R-EPOCH* rituximab, etoposide, cyclophosphamide, doxorubicin, vincristine, prednisone, *RICE* rituximab, ifosfamide, carboplatin, etoposide.

^a^Includes patients who were missing baseline LDH but were grouped regardless of its value.

The median administrations of IT prophylaxis were 4 (range 1–9), and the majority of patients received MTX alone. The dose of IT MTX and IT cytarabine was 12 and 70 mg, respectively. Patients had a median of two cycles of HD-MTX (range 1–6), at a median dose of 3500 mg/m^2^ (range 2000–3500 mg/m^2^). Overall, 23 patients (55%) had HD-MTX at the end of R-CHOP-like treatment and 19 (45%) patients during R-CHOP-like treatment. Patients receiving HD-MTX were admitted to the hospital and received leucovorin rescue starting 24 h after MTX. Overall, 6 out of 42 patients (14%) developed acute renal injury (grade 3 in all cases) related to HD-MTX; two patients at the end of chemotherapy treatment and four during systemic chemotherapy treatment. All patients recovered completely and no patient required dialysis. None of the patients received further HD-MTX after developing renal injury, two patients transitioned to IT MTX, one patient received a single administration of high-dose cytarabine, and three patients did not receive further prophylactic treatment.

### CNS relapse and the effect of prophylaxis on CNS relapse

**Fig. 2:**
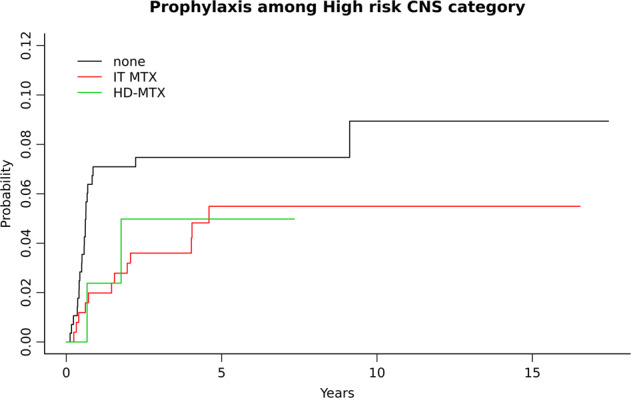
Cumulative incidence of CNS relapse rate by prophylactic strategy. IT MTX intrathecal methotrexate, HD-MTX high-dose methotrexate.

After a median follow-up of 6.8 years, 36 out of 585 patients considered HR-CNS, relapsed in the CNS with a 5-year risk of 6.5%. Fourteen (39%) patients had received prophylaxis: 12 IT and 2 HD-MTX. The risk of CNS relapse at 5 years for patients who received IT, HD-MTX, or no prophylaxis was 5.5%; 5% and 7.5% (*p* = 0.34), respectively (Fig. 2). The risk of CNS relapse by prophylaxis was similar when excluding patients who received concomitant HD-MTX and IT (Supplementary Fig. 1). CNS relapse risk was similar among patients who received R-CHOP, R-EPOCH, and R-CHOP/RICE (*p* = 0.12) (Supplementary Fig. 2). The 5-year CNS relapse risk for patients considered low-risk CNS and not included in the study was 1.1%.

**Fig. 3:**
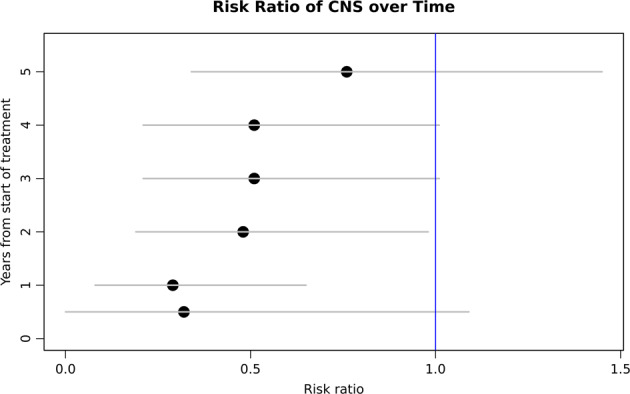
Risk ratio of CNS relapse with and without prophylaxis over time.

The median time to relapse since first diagnosis was 9 months (range 6–110 months). Patients who received prophylaxis, either IT or HD-MTX, relapsed later than patients who did not receive prophylaxis, with a median time to relapse of 19 months (range 7–55 months) vs. 8 months (range 6–110 months), respectively. The risk of relapse at 1 year was lower for patients who received prophylaxis compared to patients who did not receive prophylaxis 2% vs. 7.1%, RR 0.29 (95% CI; 0.08,0.66). However, over time, the risk of CNS relapse became closer among prophylaxis and no-prophylaxis groups, with a 3-year risk of 3.8% vs. 7.5% (RR 0.51, CI 95%; 0.22, 1.04) and a 5-year risk of 5.6% vs. 7.5% (RR 0.76, CI 95%; 0.35,1.50), respectively (Fig. 3).

At the time of CNS relapse, 26 (72%) patients presented with disease confined to the CNS and 10 (28%) patients had a concomitant systemic and CNS relapse. The brain parenchyma was the most common site of relapse (47%), followed by leptomeninges (30%) and both sites (23%). Sites of relapse by prophylactic treatment are detailed in Table 2.

**Table 2:** Site of CNS relapse by CNS prophylaxis.

	Site of CNS relapse		
CNS prophylaxis regimen	Leptomeninges	Parenchyma	Both
IT	5 (42%)	3 (25%)	4 (33%)
HD-MTX	1 (50%)	1 (50%)	–
No prophylaxis	5 (23%)	13 (57%)	4 (18%)

*CNS* central nervous system, *IT* intrathecal, *MTX* methotrexate, *HD* high-dose.

**Fig. 4:**
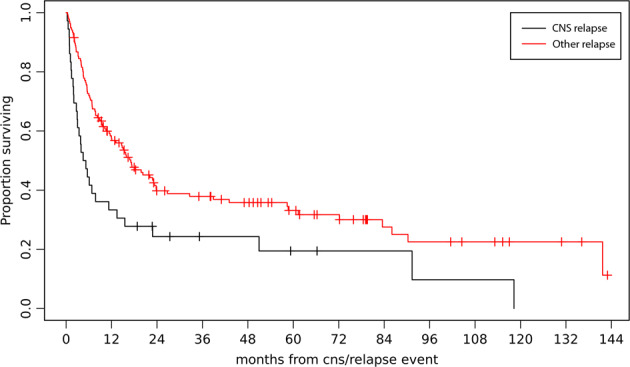
Overall survival of patients with CNS relapse vs. other relapse.

Patients with CNS relapse (*n* = 36) presented worse outcomes than patients with systemic relapse without CNS involvement (*n* = 145) with a median OS of 4.9 months vs. 17.1 months, respectively (*p* = 0.003) (Fig. 4).

**Fig. 5:**
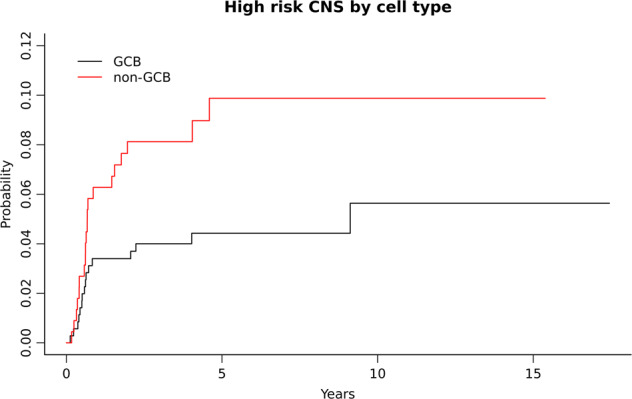
Cumulative incidence of CNS relapse by cell of origin. GCB germinal center B-cell phenotype, non-GCB non germinal center B-cell phenotype.

The COO determined by the Hans algorithm was available in 463 out of 585 patients, of whom 229 (49%) had a non-GCB phenotype and 234 (51%) patients had a GCB phenotype. Overall, 127 (55%) patients in the non-GCB group and 108 (46%) in the GCB group received prophylaxis. Patients with non-GCB phenotype had a higher risk of CNS relapse compared with patients with GCB subtype with a 5-year risk of 9.9% vs. 4.5%, respectively (*p* = 0.03) (Fig. 5).

Finally, we performed a sub-analysis excluding patients with low or intermediate CNS-IPI and bone marrow involvement (*n* = 66), since the association between bone marrow and CNS relapse is controversial. We observed a 5-year CNS relapse risk of 5.2%, 5.3%, and 7.5% for patients receiving IT and HD-MTX and no prophylaxis; respectively. Due to the low number of events in this group, we could not perform a sub-analysis to investigate the risk of CNS relapse in patients with bone marrow involvement.

## Discussion

To our knowledge, this is one of the largest series analyzing the role of CNS prophylaxis exclusively in patients with high risk of CNS relapse in the modern era. According to the CNS-IPI model, we considered high-risk for CNS relapse patients with 4 to 6 risk factors. In addition, we included patients with involvement of certain extranodal sites traditionally associated with an increased risk of CNS relapse, such as testes, breast, kidney, and bone marrow [3–6], regardless the CNS-IPI; making our results more informative. We also included double-hit lymphomas that have been associated with a high risk of CNS relapse, although estimates vary widely among different studies [2, 8, 9]. Notably, 50% of the patients included in our study did not receive CNS prophylaxis. These patients tended to be older and had worse performance status than those who receive prophylaxis. Similar results were found in recent large retrospective studies [13, 19].

Consistent with recently published studies in the rituximab era, we observed a risk of CNS relapse of 6.5% at 5 years in the HR-CNS group [13, 15, 20, 22, 23]. Furthermore, our results are similar to a recent real-world study from Sweden including 4205 patients with DLBCL, that reported a 2-year CNS risk of 8% in the high-risk patients [23]. Other investigators reported a slightly higher incidence of CNS relapse, with a 2-year risk of 10.2% [10]. The lower incidence of our study is likely due to the fact that we frequently perform brain magnetic resonance imaging in addition to a diagnostic lumbar puncture. Therefore, patients with active CNS involvement at diagnosis are promptly identified and excluded from the prophylactic strategy.

Whether CNS prophylaxis is useful in preventing CNS relapse has been addressed in several studies over the past years, including the most widely used strategy IT MTX and more recently intravenous methotrexate. The majority of the retrospective studies and post hoc analysis from prospective trials showed the lack of efficacy of IT prophylaxis in the rituximab era [10–13, 22]. In this regard, Kumar et al. using the prospectively collected National Comprehensive Cancer Network database, analyzed the risk of CNS relapse in 989 newly diagnosed DLBCL, 117 (11.8%) of whom had received prophylaxis, mostly IT MTX. They described no benefit from prophylaxis in the whole population and also in high-risk patients, though patients with involvement of kidney or adrenal glands or patients with double-hit lymphomas were not included in the high-risk group [22]. More recently, a large retrospective study including 690 patients older than 70 years of whom 271 had high-risk CNS-IPI, also described a similar rate of CNS relapse regardless the use of IT MTX prophylaxis [HR 1.34 (95% CI, 0.46–3.86)] [13]. Our series included only high-risk patients from all ages who received mostly IT MTX as prophylaxis (43%). In line with previously reported, we did not observe a clear benefit from IT MTX on preventing CNS relapse, especially after the first year of immunochemotherapy.

In recent years, the use of HD-MTX administrated mid chemotherapy cycles or after completing systemic chemotherapy was proposed as an alternative strategy to prevent CNS recurrence. The rational is based on the observation that CNS relapses frequently involve the brain parenchyma [14–16]. This approach was initially evaluated in the pre-rituximab era by the Groupe d’Etudes des Lymphomes de l’Adulte/Lymphoma Study Association comparing high-dose chemotherapy plus two cycles of HD-MTX vs. CHOP without CNS prophylaxis, with significant reduction in the CNS relapse risk of 0.8% vs. 2.7%, respectively [24]. In the modern era, different retrospective series had supported this evidence in patients treated with R-CHOP chemotherapy [14–16], although two recent retrospective studies have shown no clear benefit [17, 18]. More recently, preliminary results from a large retrospective series from Canada including 326 high-risk DLBCL patients, showed the lack of effectiveness of HD-MTX with a CNS relapse risk of 11.2% for patients who received HD-MTX (*n* = 115) vs. 12.2% for patients who did not [19]. Consistent with these studies, our data also failed to demonstrate a clinical advantage for using the more toxic intravenous HD-MTX regimen. More recently, a multicenter retrospective study found no difference in the efficacy between intravenous HD-MTX and IT MTX prophylaxis, with a CNS relapse risk of 5% vs. 7%, respectively [20].

A multicenter retrospective Australian study analyzed 217 high-risk patients treated with: (1) R-CHOP plus IT MTX, (2) R-CHOP pus HD-MTX, or (3) dose-intensive chemotherapy plus HD-MTX. They observed a lower incidence of CNS relapse in patients receiving HD-MTX compared to IT MTX, with a 3-year CNS relapse rate of 18.4% vs. 6.9% vs. 2.3%, for groups 1, 2, and 3, respectively [16]. Notably, in this study, only 37% of patients from the IT MTX group had received rituximab as part of the induction therapy, while nearly all patients in the HD-MTX group received immunochemotherapy [16]. In our study where all patients had received immunochemotherapy, we did not detect differences in the efficacy between IT and HD-MTX, although the number of patients in the HD-MTX group was low.

Although in our series the risk of CNS relapse was similar regardless the use of prophylaxis, we found that within the first year from diagnosis the risk was higher in patients who did not receive prophylaxis compared to patients who received IT or HD-MTX with a risk of 7.1% vs. 2% vs. 2.4%, respectively. However, over time, the risk became similar between groups, raising up to 5.6% and 5.2% at 5 years in the HD-MTX and IT groups, respectively. These findings were not observed in previous studies analyzing the role of CNS prophylaxis [13, 22]. However, the median follow-up in these studies was around 2.5 years, so late relapses might have been underestimated. Our evidence suggests CNS prophylaxis might help to partially control undetected CNS disease present at diagnosis delaying the occurrence of CNS relapse, rather than preventing it. Furthermore, the use of CNS prophylaxis might not prevent from late CNS relapse in the HR-CNS population.

Finally, we noticed that the presence of non-GCB phenotype determined by immunohistochemistry confers an increased risk of CNS relapse in the HR-CNS population. These results are in accordance with a recent publication analyzing the impact of COO by GEP in 1418 patients from the GOYA phase 3 trial. In this study, the authors found that ABC or unclassified phenotype was an independent risk factor for CNS relapse, and patients with high-CNS-IPI together with an ABC phenotype had a 2-year CNS relapse rate of 15% [10]. Interestingly, although we used immunohistochemistry techniques to determine the COO phenotype, we observed similar results, suggesting that in the absence of molecular analysis, COO assessed by immunohistochemistry along with other risk factors could be useful to identify high-risk patients.

In conclusion, our study highlights the need for developing more effective CNS prophylaxis regimens than MTX. Furthermore, our data in addition to emerging data from other centers, did not demonstrate clinical advantage for using intravenous HD-MTX over traditional IT MTX. Since HD-MTX is associated with higher incidence of adverse events resulting in more delays in the administration of R-CHOP cycles, we recommend that benefit/risk assessment should be carefully considered before adopting HD-MTX for CNS prophylaxis.

## Supplementary information

Supplemental material
