# Supplementary material for: Prophylaxis with intrathecal or high-dose methotrexate in diffuse large B-cell lymphoma and high risk of CNS relapse
Source: Blood Cancer J. 2021 Jun 16;11(6):113. doi: 10.1038/s41408-021-00506-3 (PMC8209097; doi:10.1038/s41408-021-00506-3)
Supplement: Supplementary file 1 — Supplemental material [file 41408_2021_506_MOESM1_ESM.docx]

Supplementary material:

Supplemental Table 1. CNS relapses in patients with low or intermediate CNS-IPI and involvement of extranodal sites not considered HR-CNS relapse and not included in the analysis.

|  | N | CNS relapse |
| --- | --- | --- |
| Low or intermediate CNS IPI |  |  |
| Paraspinal mass | 38 | 0 |
| Sinus | 69 | 3 |
| Orbit | 6 | 0 |
| Skull | 6 | 1 |

Supplemental_Figure 1. Cumulative incidence of CNS relapse by prophylaxis excluding patients who received concomitant IT and HD-MTX prophylaxis (n=574).

Supplemental_Figure 2. CNS rate by immunochemotherapy regimen.

p=0.125
